# Cytokine Profile in Plasma Extracellular Vesicles of Parkinson’s Disease and the Association with Cognitive Function

**DOI:** 10.3390/cells10030604

**Published:** 2021-03-09

**Authors:** Lung Chan, Chen-Chih Chung, Jia-Hung Chen, Ruan-Ching Yu, Chien-Tai Hong

**Affiliations:** 1 Department of Neurology, Shuang Ho Hospital, Taipei Medical University, New Taipei City 23561, Taiwan; cjustinmd@gmail.com (L.C.); 10670@s.tmu.edu.tw (C.-C.C.); gary.320@hotmail.com (J.-H.C.); 2 Department of Neurology, School of Medicine, College of Medicine, Taipei Medical University, Taipei 11031, Taiwan; 3 Graduate Institute of Biomedical Informatics, Taipei Medical University, Taipei 11031, Taiwan; 4 Division of Psychiatry, University College London, London W1T 7NF, UK; ruan-ching.yu.18@ucl.ac.uk

**Keywords:** extracellular vesicles, Parkinson’s disease, inflammation, cytokine

## Abstract

Plasma extracellular vesicles (EVs) containing various molecules, including cytokines, can reflect the intracellular condition and participate in cell-to-cell signaling, thus emerging as biomarkers for Parkinson’s disease (PD). Inflammation may be a crucial risk factor for PD development and progression. The present study investigated the role of plasma EV cytokines as the biomarkers of PD. This cross-sectional study recruited 113 patients with PD, with mild to moderate stage disease, and 48 controls. Plasma EVs were isolated, and the levels of cytokines, including pro-interleukin (IL)-1β, IL-6, IL-10, tumor necrosis factor (TNF)-α, and transforming growth factor (TGF)-β1, were evaluated. Patients with PD had significantly increased plasma EV pro-IL-1β and TNF-α levels compared with controls after adjustment for age and sex. Despite the lack of a significant association between plasma EV cytokines and motor symptom severity in patients with PD, cognitive dysfunction severity, assessed using the Mini-Mental State Examination (MMSE) and Montreal Cognitive Assessment, was significantly associated with plasma EV pro-IL-1β, IL-6, IL-10, and TNF-α levels. This association was PD specific and not found in controls. Furthermore, patients with PD cognitive deficit (MMSE < 26) exhibited a distinguished EV cytokine profile compared to those without cognitive deficit. The findings support the concept of inflammatory pathogenesis in the development and progression of PD and indicate that plasma EV cytokines may serve as PD biomarkers in future.

## 1. Introduction

Parkinson’s disease (PD) is the second most common neurodegenerative disease [1], and its prevalence is expected to increase with the aging of society [2]. Local inflammation of the brain (neuroinflammation) is a crucial environmental stress for the loss of dopaminergic neurons in PD. Activated microglia and immune responses were found in the substantia nigra on the postmortem examination of individuals with PD [3,4,5,6]. After the uptake of excessive α-synuclein from dopaminergic neurons, microglial cells activate and then release proinflammatory cytokines, triggering inflammatory stress, which may induce neuronal apoptosis [7]. In addition, systemic inflammation contributes to neuroinflammation; epidemiological studies have indicated a strong association between inflammatory bowel diseases and PD [8] and demonstrated the protective effect of nonsteroidal anti-inflammatory drugs on PD [9]. The concept of gut–brain axis also supports the role of systemic inflammation in PD. Gut dysbiosis causes leaky gut syndrome, which allows proinflammatory lipopolysaccharides (LPSs) to enter the circulation and induce inflammation in the peripheral nervous system and brain simultaneously [10].

Inflammation is tightly regulated by cytokines. Cytokines secreted by the immune system serve as paracrine or endocrine signals to downstream cellular and environmental conditions. Cytokines can be proinflammatory, including interleukin (IL)-1β, tumor necrosis factor (TNF)-α, and IL-6, or anti-inflammatory, including IL-10 and transforming growth factor (TGF)-β, and a balance between the two regulates the inflammatory reaction in the body [11]. Elevated proinflammatory cytokines have been detected in the peripheral blood of patients with PD, consolidating the link between PD and inflammation. However, the results are highly variable among study groups, thus limiting the application of proinflammatory cytokines as the biomarkers of PD [12]. Furthermore, the extremely short half-life of free-form cytokines in peripheral blood and stress-related fluctuations in cytokine secretion introduce more variability and inconsistency in the blood cytokine profile [13].

Instead of their soluble free form, a fraction of cytokines in peripheral blood are present inside extracellular vesicles (EVs) [14]. Secreted by nearly all somatic cells, EVs are small vesicles enclosed in a lipid membrane. The content, or cargo, of EVs consists of lipids, small molecules, nucleic acids, and proteins—specifically proteins associated with the plasma membrane, cytosol, and those involved in lipid metabolism [15]. EVs help in remote cell-to-cell signal transmission and cross the blood–brain barrier (BBB) without damaging the structure [16]. Through cytokines present in EVs, donor cells can modulate the inflammatory reaction in recipient cells [17]. The blood EV cytokine profile likely reflects systemic inflammation and may be superior to the free-form counterparts because of the stability of EV-associated cytokine, leading to longer half-life [18] and avoiding the short-term stress-associated soluble free-from cytokine surge in the blood [13].

Although the blood EV cytokine profile has been examined in some malignant and infectious diseases [19,20], it has not been investigated in PD. The present study examined whether the blood EV cytokine profile can serve as a biomarker to distinguish individuals with and without PD and be associated with PD symptoms.

## 2. Materials and Methods

### 2.1. Study Participants

A total of 161 participants (113 patients with PD and 48 controls) were enrolled in this study. PD was diagnosed on the basis of the UK Parkinson’s Disease Society Brain Bank Clinical Diagnostic Criteria [21]. Only patients with mild to moderate PD (stages 1 to 3 PD, according to the Hoehn and Yahr scale) were included in the PD group. Controls included in this study did not have any known neurodegenerative, psychiatric, or major systemic diseases (malignant neoplasm and chronic kidney disease) and were regularly followed up in outpatient clinics for chronic conditions (hypertension, diabetes, or hyperlipidemia). This study was approved by the Joint Institutional Review Board of Taipei Medical University (approval no. N201609017 and N201801043).

### 2.2. Clinical Assessments

All participants were interviewed to obtain baseline demographic data. Their cognitive function was investigated by trained nurses by using the Taiwanese versions of the Mini-Mental State Examination (MMSE) and Montreal Cognitive Assessment (MoCA). Participants with an MMSE score of < 26 were considered to have cognitive deficit. All patients with PD were evaluated using parts I–III of the Unified Parkinson’s Disease Rating Scale (UPDRS) during an outpatient visit. The time between the most recent dose of anti-PD medication and the assessment of UPDRS part IV was not recorded; patients with PD were assumed to be on their “on” time.

### 2.3. Plasma EV Isolation and Validation

For the isolation of plasma EVs, venous blood samples were collected from all study participants. Whole blood was centrifuged at 13,000× *g* for 20 min to isolate plasma. Next, 1 mL of plasma was passed through an exoEasy Maxi kit (Qiagen, Venlo, NL, Cat.#76064, for exosome isolation according to the manufacturer’s instructions. The last step of the isolation was the elution of the exosome from the column. Usually, 400 μL of the eluent was obtained.

Isolated EVs were validated by detecting the presence of tetraspanins (CD9, CD81 and CD63), the presence of EV inner component (Tumor susceptibility gene 101), and negative of mitochondrial protein (cytochrome c), and their sizes were determined through nanoparticle tracking with a peak around 100nM. The validation details have been described in previous studies from our PD cohort [22,23].

### 2.4. Western Blot Analysis of Plasma EV Cytokine

Isolated plasma EVs were directly lysed using protein sample buffer (RIPA Lysis Buffer, Millipore, MA, US) and analyzed using a protein sodium dodecyl sulfate and polyacrylamide gel. Antibodies against IL-1β (Cell Signaling Technology, Danvers, MA, USA, Cat.#12242, 1:1000), IL-6 (Cell Signaling Technology, Danvers, MA, USA, Cat.#12912, 1:1000), TNF-α (Cell Signaling Technology, Danvers, MA, USA, Cat.#11948, 1:1000), TGF-β1 (Abcam, Cambridge, UK, Cat.ab215715, 1:1000), and IL-10 (Abcam, Cambridge, UK, Cat.ab133575, 1:1000) were used. Regarding the IL-1β, according the manufacture’s information, both precursor (31 kD) and mature form of IL-1β (17 kD) are detectable. However, we only managed to identify one clear sharp band in the western blot between 25 kD to 37 kD, which is speculated to be the precursor (pro-IL-1β) but negative the presence of any identifiable signal around 17 kD (mature IL-1β). Antibodies were prepared in tris-buffered saline containing 0.1% Tween 20 (TBST) and 5% bovine serum albumin (BSA). Secondary antibodies, including anti-mouse IgG conjugated with horseradish peroxidase (HRP) (115-035-003) and anti-rabbit IgG conjugated with HRP (111-035-003), were purchased from Jackson ImmunoResearch (West Grove, PA, USA). Protein blot intensities were quantified using Image J software. Cytokine levels were normalized to the heat shock protein (HSP)-70 level (Proteintech, Rosemont, IL, USA, Cat.10995-1-AP, 1:2000). The rationale to select HSP-70 as the control was based on the consideration that the cytokines we investigated are located in the intravesicular space and that the levels of HSP-70 inside the EV are relative steady. To ensure that data could be compared between different gels, all data were normalized to the average of the control group in the same gel.

### 2.5. Statistical Analysis

All statistical analyses were performed using SPSS for Windows 10 (version 19; SPSS, Chicago, IL, USA). The chi-square test was performed to compare the sex distribution between patients with PD and controls. The nonparametric Mann-Whitney U test was used to compare the plasma EV levels of cytokines and other continuous variables between patients with PD and controls and between patients with PD with and without cognitive deficit. Spearman’s rank correlation was used to assess the association between the plasma EV cytokine level and the UPDRS score and cognitive tests. Multivariable logistic regression was applied to investigate the association between plasma EV cytokine and PD in overall study participants and the cognitive function in patients with PD after adjusting for age and sex. A *p* value of <0.05 was considered statistically significant.

## 3. Results

Table 1 lists the participants’ baseline demographic data. No differences in age and sex were observed between patients with PD and controls. Since the control group did not have a history of dementia and other neurodegenerative diseases, significant cognitive function differences between the groups were noted, as indicated by the Mini-Mental State Examination (MMSE) and Montreal Cognitive Assessment (MoCA) scores. The mean PD duration was 2.71 ± 2.48 years, and the mean Unified Parkinson’s Disease Rating Scale (UPDRS) scores in parts I, II, and III were 2.48 ± 2.00, 7.92 ± 5.82, and 22.48 ± 9.85, respectively.

**Figure 1:**
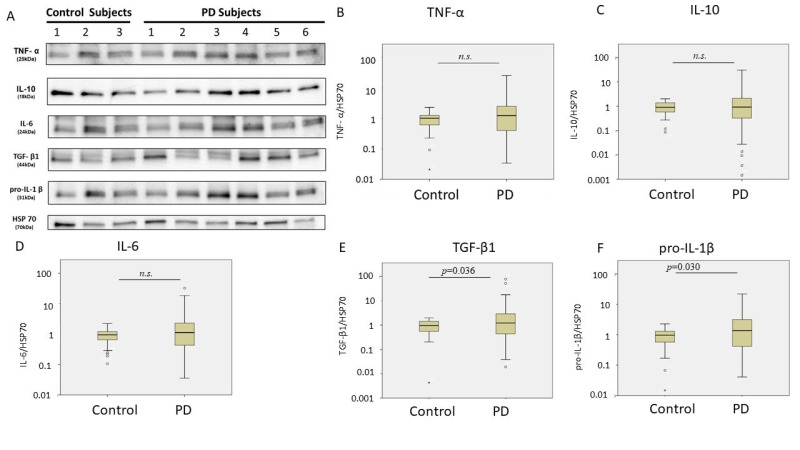
Cytokine levels in plasma extracellular vesicles in patients with Parkinson’s disease (PD) (*n* = 113) and controls (*n* = 48). (**A**) Representative protein blot images of different cytokines, including pro-interleukin (IL)-1β, IL-6, IL-10, tumor necrosis factor (TNF)-α, and transforming growth factor (TGF)-β1. Heat shock protein (HSP)-70 was the protein loading control. (**B**–**F**) Quantification data of western blot to compare the TNF-α, IL-10, IL-6, TGF-β1, and pro-IL-1β levels between control and PD subjects. The box-and-whisker plot was presented as median, 1st quartile and 3rd quartile of the box, the 95th and 5th percentile for the upper and lower whisker, ○ for the outliners. *n.s.*, nonsignificant.

In plasma EV, patients with PD had significantly higher pro-IL-1β (median: 1.37-fold increase after normalization by the control) and TGF-β1 (median: 1.22-fold increase after normalization by the control) levels than did the controls (Figure 1). After adjustment for age and sex, the diagnosis of PD was significantly associated with pro-IL-1β and TNF-α levels (Table 2).

We further investigated the association between plasma EV cytokines and the severity of motor and cognition in patients with PD. The levels of most plasma EV cytokines were not associated with UPDRS part I, II, or III scores, except for the pro-IL-1β level, which was associated with UPDRS-II, and the TGF-β1 level, which was associated with UPDRS-III. By contrast, the levels of all plasma EV cytokines, except IL-6, were significantly associated with the MMSE score. In addition, pro-IL-1β and TGF-β1 levels were significantly correlated with the MoCA score in patients with PD (Table S1 and Figure S1). After adjustment for age and sex, plasma EV pro-IL-1β, IL-6, TNF-α, and IL-10 levels were associated with MMSE and MoCA scores (Table 3). This association between cognition and plasma EV cytokine levels was not observed in the control group (Table S2).

**Figure 2:**
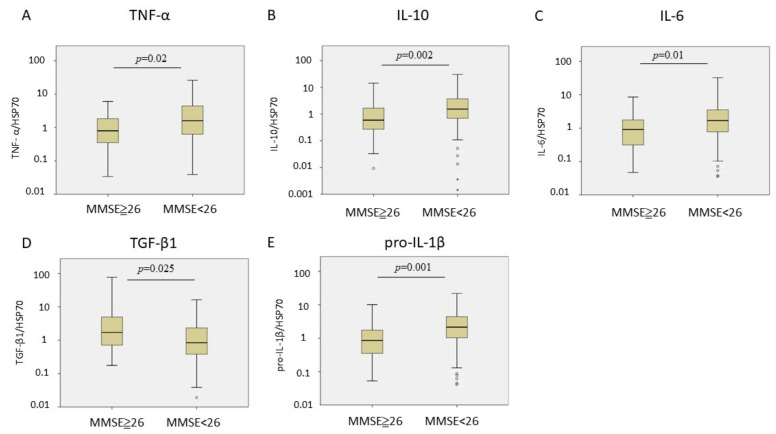
Cytokine levels in plasma extracellular vesicles for Parkinson’s disease with Mini-Mental State Examination (MMSE) less than 26 (*n* = 51) and equal/above 26 (*n* = 62). (**A**–**E**) Comparison of tumor necrosis factor (TNF)-α, interleukin (IL)-10, IL-6, transforming growth factor (TGF)-β1, and pro-IL-1β levels between PD patients with MMSE less than 26 and equal/above 26. Heat shock protein (HSP)-70 was the protein loading control. The box-and-whisker plot was presented as median, 1st quartile and 3rd quartile of the box, the 95th and 5th percentile for the upper and lower whisker, ○ for the outliners.

Finally, patients with PD and cognitive deficit (MMSE < 26) (*n* = 51) exhibited significantly increased plasma EV pro-IL-1β, IL-6, TNF-α, and IL-10 levels and a significantly decreased TGF-β1 level (Figure 2). After adjustment for age and sex, PD-cognitive deficit was positively associated with plasma EV pro-IL-1β, IL-6, TNF-α, and IL-10 levels and negatively with the TGF-β1 level (Table 4).

## 4. Discussion

The results of the present study demonstrated that the levels of plasma EV proinflammatory cytokines pro-IL-1β and TNF-α were elevated in patients with PD compared with controls after adjustment for age and sex. Moreover, the plasma EV cytokine profile was significantly associated with cognitive function in patients with PD but not in controls. Furthermore, a significant difference in the plasma EV cytokine profile was noted between patients with PD with and without cognitive deficit (MMSE < 26). This is the first study to investigate the association between PD and the plasma EV cytokine profile, which may be used as a biomarker in PD. The findings also indicate that inflammation plays an essential role in the development and progression of PD.

Immune cells can release cytokines and regulate inflammation through EVs [18]. The EV cytokine profile is not identical to the release of free-form cytokines, and different stimulation of immune cells results in a distinct EV cytokine profile [24]. EV-associated cytokines preserve their functionality in recipient cells. Physiologically, macrophage-derived exosomes, a type of EV with a size ranging from 50 to 150 nm, contain IL-6 and IL-8 and stimulate placental cytokine release during pregnancy [25]. Pathologically, tumor-derived EVs contain abundant TGF-β, promoting tumor progression by enhancing recipient tumor cell migration [26]. EV-associated cytokines can also be used therapeutically, i.e., IL-10-loaded EVs could significantly attenuate ischemic/reperfusion injury-related renal tubular injury and inflammation [27]. Clinically, the plasma exosome cytokine profile has been demonstrated to be associated with diseases. In people with human immunodeficiency virus (HIV) infection, a marked elevation of most cytokines is noted in plasma exosomes compared with the control group and the free-form soluble cytokines from the patients’ blood [20]. In patients with gastric cancer, exosomal TNF-α and TGF-β levels were increased, whereas the IL-10 level was decreased [19]. Taken together, the findings suggest that the blood EV cytokine profile is useful as a biomarker for inflammation-associated diseases.

Considering the role of inflammation in PD pathogenesis, investigating the blood cytokine profile as a disease marker is a nonstop journey. However, the results are highly variable, thus limiting their clinical application. A meta-analysis of 25 studies including 2654 participants demonstrated significantly higher IL-6, TNF, IL-1β, IL-2, and IL-10 levels in patients with PD. However, all cytokines, except IL-10, exhibited considerable heterogeneity among the pooled studies, with I^2^ > 50% [12]. Age, sex, disease duration, disease severity, medication, and cytokine assessments (use of the enzyme-linked immunosorbent assay or not) accounted for the high heterogeneity. Moreover, the short half-life and fluctuation of free-form cytokines in peripheral blood are more confounding. Since EV cytokines constitute a comparable level of cytokines with the free-from cytokine in the blood and are associated with diseases, they may serve as promising new biomarkers to investigate the association between PD and inflammation. The stability of EVs prevents the fluctuation in cytokine levels due to transient surge or degradation and thus may more precisely reflect the steady state of systemic inflammation. The results of the present study revealed that the levels of pro-IL-1β and TNF-α-containing plasma EVs were elevated in patients with PD. IL-1β is a potent proinflammatory cytokine that stimulates CD4+T cells and promotes differentiation into T helper cell lineages [28]. LPS increased the cellular secretion of IL-1β-containing microvesicles [29]. The elevation of plasma pro-IL-1β-containing EVs may represent the activation of the systemic inflammatory signal. TNF-α, a proinflammatory cytokine, regulates several complex signaling pathways, including apoptosis, survival, inflammation, and cellular differentiation [30]. Upon cellular stress, such as HIV infection, pro-TNF-α is more likely to be transferred into lipid rafts and form EVs for secretion [31]. The association of diseases with other cytokines—IL-6, IL-10, and TGF-β—in EVs remains poorly studied. However, in the present study, the cognition of patients with PD was associated with most plasma EV cytokines, thereby indicating the role of inflammation in PD progression. The lack of a similar association in control participants indicated a PD-specific association between cognition and the plasma EV cytokine profile.

This study is the first to investigate the plasma EV cytokine profile in patients with PD, thus providing a novel platform to assess inflammation in these patients. Although any single cytokine fails to distinguish patients with PD from controls, evaluating them as a panel and analyzing them by using an artificial intelligence-assisted artificial neural network could effectively predict the neurological outcome after stroke [23,32]. Moreover, the significantly different plasma EV cytokine profiles in patients with PD with and without cognitive deficit suggest the role of inflammation in PD progression. Considering the lack of available disease modification treatments for patients with PD and PD patients with dementia nowadays, the manipulation of inflammation may be a promising target of therapy, and the plasma EV cytokine profile can serve as the biomarker of the therapeutic effect.

The present study also has some limitations. The cytokine assessment with Western blotting was a semiquantitative method that lacked absolute concentration for further clinical application and direct comparison with other studies. Meanwhile, the present study did not investigate the soluble free-form cytokine simultaneously, which limits detailed understanding about the blood cytokine fraction in PD patients. In addition, the cross-sectional design precluded the definitive conclusion that the association between cognition and the plasma EV cytokine profile in patients with PD resulted from disease progression. However, the lack of a similar association in control participants indicates that the relationship is PD specific. Another limitation is that the inflammatory profile in peripheral blood may not directly reflect neuroinflammation in the brain due to the presence of the BBB. Nevertheless, neurodegeneration in PD usually accompanies the breakdown of the BBB and the increase of permeability [33], which may cause neuroinflammation to be reflected in peripheral blood. Moreover, EVs can penetrate the BBB [34], which further reduces concern regarding the lack of representation of peripheral blood to the brain. Regarding the cohort itself, the disparity of PD patients and controls is remarkable, which may result from the lack of willing participation in the controls. Some of the patients with early stage PD had low MMSE or MoCA scores because of low education levels. The extension of mandatory education from six to nine years in Taiwan in 1968 means that persons older than 64 years old were most likely to have had a six-year basic education, thus undermining their cognitive capability in the tests.

In conclusion, the findings of the present study revealed that plasma EV pro-IL-1β and TNF-α levels in patients with PD were higher than those in controls after adjustment for age and sex. In patients with PD, the plasma EV cytokine profile was associated with cognition, and patients with PD cognitive deficit exhibited a distinct pattern of the plasma EV profile compared with those without cognitive deficit. Thus, plasma EV cytokines may be promising as biomarkers of PD. The findings also support the concept of inflammation as a pathogenetic mechanism in PD. A longitudinal follow-up study is essential to investigate alterations in the plasma EV cytokine profile with disease progression.

## Figures and Tables

**Table 1:** Demographic data in Parkinson’s disease (PD) patients and control group.

	Control	PD	*p* Value
Number of patients	48	113	-
Age (years)	67.94 ± 7.50	69.66 ± 8.41	0.214
Female	39	27	0.124
Disease duration (years)	-	2.71 ± 2.48	-
MMSE	27.36 ± 2.77	24.18 ± 6.36	<0.001
MoCA	23.25 ± 3.89	20.43 ± 6.02	0.003
UPDRS-I	-	2.48 ± 2.00	-
UPDRS-II	-	7.92 ± 5.82	-
UPDRS-III	-	22.48 ± 9.85	-

MMSE, Mini-Mental State Examination; MoCA, Montreal Cognitive Assessment; UPDRS, Unified Parkinson’s Disease Rating Scale. Data was presented as mean ± standard deviation.

**Table 2:** Association of plasma extracellular vesicle cytokine levels with the diagnosis of Parkinson’s disease after adjustment for age and sex.

	Std. β	*p* Value	95% CI	R^2^ of the Model
IL-6	0.150	0.056	−0.03~2.26	0.067
pro-IL-1β	0.206	0.008	0.32~2.20	0.087
TNF-α	0.175	0.025	0.16~2.40	0.071
TGF-β1	0.120	0.133	−0.62~4.72	0.075
IL-10	0.147	0.061	−0.06~2.67	0.087

Std., standard; β, coefficient; CI, confidence interval.

**Table 3:** Association of plasma extracellular vesicle cytokine levels with the cognition in Parkinson’s disease patients after adjusting for age and sex.

MMSE					MoCA			
	Std. β	*p* Value	95% CI	R^2^ of the Model	Std. β	*p* Value	95% CI	R^2^ of the Model
IL-6	−0.217	0.024	−0.27~−0.02	0.095	−0.286	0.004	−0.29~−0.06	0.133
Pro-IL-1β	−0.284	0.003	−0.25~−0.05	0.123	−0.308	0.002	−0.26~−0.06	0.133
TNF-α	−0.227	0.017	−0.26~−0.03	0.123	−0.300	0.002	−0.30~−0.07	0.168
TGF-β1	0.121	0.215	−0.07~0.30	0.054	0.153	0.132	−0.04~0.34	0.061
IL-10	−0.239	0.012	−0.33~−0.04	0.116	−0.237	0.017	−0.33~−0.03	0.111

MMSE, Mini-Mental State Examination; MoCA, Montreal Cognitive Assessment; Std., standard; β, coefficient; CI, confidence interval.

**Table 4:** Association of plasma extracellular vesicle cytokine levels with the presence of Parkinson’s disease cognitive deficit (Mini-Mental state Examination (MMSE) less than 26) after adjustment for age and sex.

	Std. β	*p* Value	95% CI	R^2^ of the Model
IL-6	0.209	0.031	0.15~3.13	0.090
pro-IL-1β	0.260	0.007	0.47~2.90	0.110
TNF-α	0.225	0.019	0.29~3.20	0.114
TGF-β1	−0.198	0.039	−7.04~−0.18	0.083
IL-10	0.240	0.013	0.50~4.07	0.111

Std., standard; β, coefficient; CI, confidence interval.

## Data Availability

The data generated during the current study are available from the corresponding author on reasonable request.

## Supplementary Materials

The following are available online at https://www.mdpi.com/2073-4409/10/3/604/s1, Figure S1: Association between plasma extracellular vesicle cytokines with the cognitive scale in patients with Parkinson’s disease; Table S1: Correlations between plasma extracellular vesicle cytokines with various clinical assessment scores in patients with Parkinson’s disease; Table S2: Correlations between plasma extracellular vesicle cytokines with cognition in control participants after adjustment for age and sex.
