# Supplementary material for: Cytokine Profile in Plasma Extracellular Vesicles of Parkinson’s Disease and the Association with Cognitive Function
Source: Cells. 2021 Mar 9;10(3):604. doi: 10.3390/cells10030604 (PMC7999703; doi:10.3390/cells10030604)
Supplement: Supplementary file 1 [file cells-10-00604-s001.pdf]

Supplementary Information

Table S1. Correlations between plasma extracellular vesicle cytokines with various clinical assessment scores in patients with Parkinson's disease.

	UPDRS-I	UPDRS-II	UPDRS-III	MMSE	MoCA
IL-6	−0.008	0.135	0.015	−0.170	−0.128
pro-IL-1β	0.051	0.176*	0.045	−0.250**	−0.203*
TNF-α	−0.066	0.112	−0.015	−0.190*	−0.145
TGF-β1	−0.095	−0.083	0.195*	0.279**	0.200*
IL-10	−0.018	0.103	−0.050	−0.196*	−0.146

MMSE, Mini-Mental State Examination; MoCA, Montreal Cognitive Assessment; UPDRS, Unified Parkinson's Disease Rating Scale. *, $p < 0.05$, **, $p < 0.01$.

Table S2. Correlations between plasma extracellular vesicle cytokines with cognition in control participants after adjustment for age and sex.

	MMSE			MoCA		
	Std.β	*p* value	95% CI	Std.β	*p* value	95% CI
IL-6	−0.006	0.969	−0.11 to 0.01	−0.010	0.95	−0.05 to 0.05
pro-IL-1β	−0.103	0.524	−0.14 to 0.07	−0.031	0.848	−0.06 to 0.05
TNF-α	0.036	0.820	−0.09 to 0.11	0.155	0.324	−0.02 to 0.07
TGF-β1	−0.166	0.300	−0.16 to 0.05	0.235	0.142	−0.01 to 0.08
IL-10	−0.134	0.394	−0.15 to 0.06	−0.131	0.408	−0.07 to 0.03

MMSE, Mini-Mental State Examination; MoCA, Montreal Cognitive Assessment; Std, standard; CI, confidence interval.

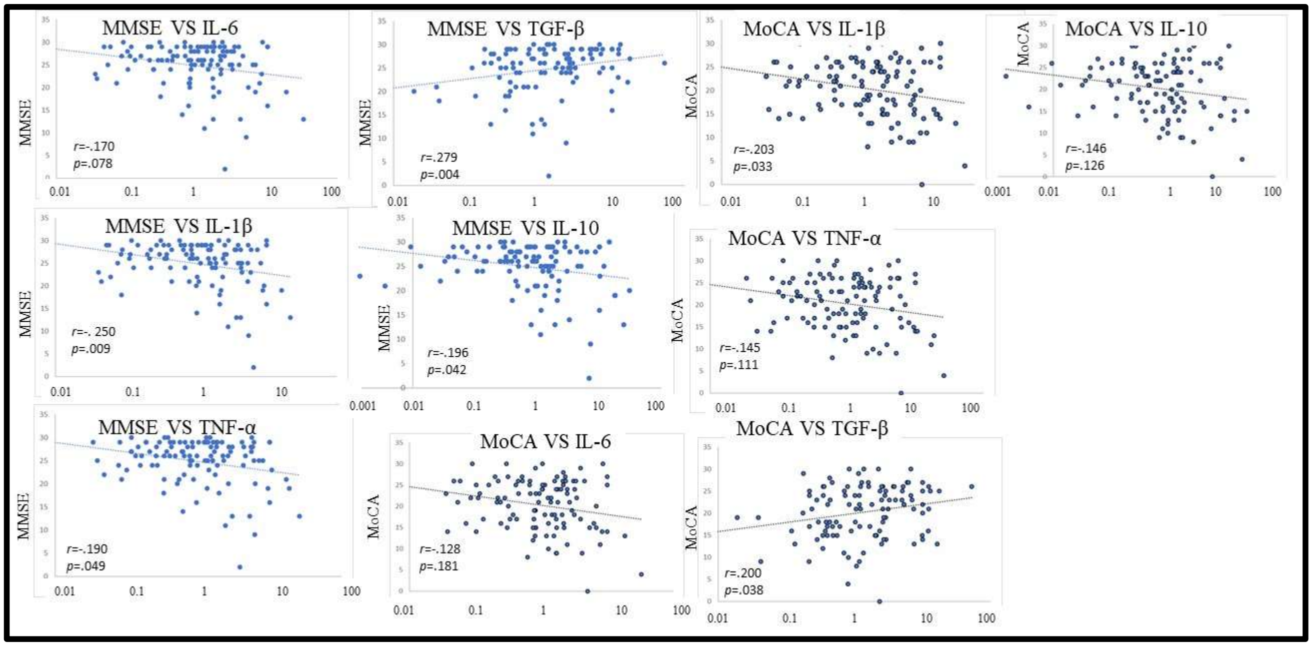

Figure S1. Association between plasma extracellular vesicle cytokines with the cognitive scale in patients with Parkinson's disease.
